# Comparing quality indicator rates for home care clients receiving palliative and end-of-life care before and during the Covid-19 pandemic

**DOI:** 10.1186/s12904-023-01336-9

**Published:** 2024-01-05

**Authors:** Julia Kruizinga, Kathryn Fisher, Dawn Guthrie, Melissa Northwood, Sharon Kaasalainen

**Affiliations:** 1 McMaster University, 1280 Main Street West, Hamilton, ON Canada; 2 School of Nursing, McMaster University, 1280 Main Street West, Hamilton, ON Canada; 3 Department of Kinesiology and Physical Education, Wilfrid Laurier University, Waterloo, ON Canada; 4 Department of Health Sciences, Wilfrid Laurier University, Waterloo, ON Canada

**Keywords:** Palliative and end-of-life care, Home care, Quality indicators

## Abstract

**Background:**

The consensus among Canadians with regards to end-of-life preferences is that with adequate support the majority prefer to live and die at home.

**Purpose:**

To compare quality indicator (QI) rates for home care clients receiving palliative and end-of-life care prior to and after the onset of the COVID-19 pandemic.

**Methods:**

A retrospective population-based cohort design was used. Sixteen QIs informed by existing literature and a preliminary set of QIs recently evaluated by a modified Delphi panel were compared. Data were obtained from the interRAI Palliative Care instrument for Ontario home care clients for two separate cohorts: the pre-COVID (January 14, 2019 to March 16, 2020) and COVID cohort (March 17, 2020 to May 18, 2021). A propensity score analysis was used to match (using nearest neighbour matching) on 21 covariates, resulting in a sample size of 2479 unique interRAI Palliative Care assessments in each cohort. Alternative propensity score methods were explored as part of a sensitivity analysis.

**Results:**

After matching the pre-COVID and COVID cohorts, five of the 16 QIs had statistically significant differences in the QI rates (change from pre-COVID to COVID): decrease in prevalence of severe or excruciating daily pain (*p* = 0.03, effect size=-0.08), decrease in prevalence of caregiver distress (*p* = 0.02, effect size=-0.06), decrease in prevalence of negative mood (*p* = 0.003, effect size=- 0.17), decrease in prevalence of a delirium-like syndrome (*p* = 0.001, effect size=-0.25) and decrease in prevalence of nausea or vomiting (*p* = 0.04, effect size=-0.06). While the alternative propensity score methods produced slightly different results, no clinically meaningful differences were seen between the cohorts when effect sizes were examined. All methods were in agreement regarding the highest QI rates, which included the prevalence of shortness of breath with activity, no advance directives, and fatigue.

**Conclusion:**

This study is the first to examine differences in QI rates for home care clients receiving palliative and end-of-life care before and during COVID in Ontario. It appears that QI rates did not change over the course of the pandemic in this population. Future work should be directed to understanding the temporal variation in these QI rates, risk-adjusting the QI rates for further comparison among jurisdictions, provinces, and countries, and in creating benchmarks for determining acceptable rates of different QIs.

**Supplementary Information:**

The online version contains supplementary material available at 10.1186/s12904-023-01336-9.

## Background

Home care is designed to promote well-being and independence for recipients while building health system capacity [1]. An essential component of home care is palliative and end-of-life care (PEoLC). PEoLC can be defined as care that promotes quality of life, including the dimensions of symptom management, social, spiritual, and psychological needs and often during the last six months to one year of life [2]. The consensus among Canadians with regards to PEoLC preferences is that with adequate support the majority prefer to live and die at home [3, 4]. PEoLC home care services have been shown to be associated with outcomes of improved symptom management [5], increased likelihood of having a home death [6], improved caregiver burden [7], and satisfaction with healthcare [8] for persons with life-limiting illnesses [9]. However, the quality of palliative and end-of-life *home care* has been reported to vary substantially across geographical regions and by type of illness [10, 11]. With an increasing focus on the home care sector due to a rapidly aging population with increasing PEoLC needs, better quality of and access to PEoLC is a major priority for Canadians [12–15].

Health systems and services have been significantly disrupted because of the corona virus disease (COVID) pandemic which began in 2019, highlighting existing and ongoing challenges in the delivery of quality home-based care for individuals with serious or life-limiting illnesses [16]. For example, home care coordinators and other practitioners were unable to do home visits and care delivery shifted to virtual formats [17–19] where there were limitations in developing therapeutic relationships, assessing illness severity, and with connectivity and training, particularly in rural and remote areas [20]. During the COVID-19 pandemic the quality of PEoLC within home care has received little attention when compared to the hospital and long-term care sectors and represents a critical time period to evaluate [20]. Home-based care is critical and has been emphasized throughout the pandemic given the potential for surging rates of hospital admissions related to COVID-19 during each wave [21], as well as its role in essential pain and symptom management, advance care planning, and grief and bereavement supports [22, 23]. Despite the call for quality improvement in the home care sector, a quality assurance mechanism to measure, monitor, and benchmark across jurisdictions is lacking. Progress in quality measurement and evaluation is a crucial step necessary to inform healthcare system decision-making and wide-spread planning of PEoLC and to identify areas to improve the quality of PEoLC for individuals living at home [10, 24]. Quality indicators (QIs) are defined as “quantitative measures that provide information about the effectiveness, safety and/or people-centredness of care” (23, pg. 33) and are one way to gauge how a system is performing [25]. Therefore, the **purpose of this study** was to compare QI rates for home care clients receiving palliative and end-of-life care prior to and after the onset of the COVID-19 pandemic.

## Methods

### Study design

A retrospective population-based cohort design [26, 27] was used to compare QI rates generated from interRAI PC assessment data for home care clients designated to receiving PEoLC in Ontario, Canada prior to and during the COVID-19 pandemic. Retrospective analysis is an efficient and time-effective approach for monitoring and evaluating PEoLC, particularly over time periods and to compare across jurisdictions and demographic groups [27].

### Data sources

The interRAI PC is a standardized clinical assessment instrument used to inform care planning and is designed for adults aged 18 and older with end-of-life (EOL) needs [28]. It was developed by a multinational research consortium as part of a suite of interRAI instruments and while it is not mandated for use in Ontario, it is frequently used for those receiving PEoLC [29]. To the best of our knowledge, all 14 regions were using the interRAI PC on intake into their palliative home care program. Over time, some regions have opted not to use it as a re-assessment tool. The interRAI PC assessment is typically done on admission to the home care program, for routine re-assessment, and at a time of the questions in the significant change, such as after hospitalization. The interRAI PC instrument has established validity and reliability for its measures [30], where the eight domains (symptoms/conditions, cognitive competency and communication, mood, functional status, preferences, social relations, spirituality, services and treatments) have an average kappa ranging from 0.76 to 0.95. A semi-structured interview process is used to obtain information on clients’ strengths, needs, and preferences to primarily guide care planning and service delivery. interRAI PC assessments are completed by trained professionals (usually a registered nurse) using a software application and these electronic assessments are shared with the relevant agency providers as part of the home care health record [31]. Data are self-reported by home care clients and/or their caregiver along with trained professionals who conduct the assessment and verify the data. The data are anonymized by Ontario Health Shared Services, and then stored at the University of Waterloo on a secure server for use by interRAI Canada Fellows and their students for research purposes.

### Cohort periods

The COVID-19 pandemic was announced as a state of emergency by the Ontario provincial government on March 17th, 2020 [32]. The time periods for each cohort were selected based on when COVID was initially declared a pandemic in Ontario and for the duration of time data were available for this study. interRAI PC data were available until May 18th, 2021 when analysis began and so a timeframe of 60 weeks was available and applied to the COVID cohort. The same timeframe was applied to the pre-COVID cohort.

### Study cohort

This study involved the comparison of two groups of home care clients aged 18 years and older receiving PEoLC in Ontario, Canada. The first group (pre-COVID) was home care clients with an interRAI PC assessment between January 1st 2019 to March 16th 2020, and the second group (COVID) were similarly assessed between March 17th 2020 to May 18th 2021. The two cohorts were further restricted to follow-up assessments (> 30 days) as admission assessments likely do not reflect the quality of care during the time of the assessment [33]. Where duplicates (individuals in both cohorts) existed, clients were assigned to the COVID cohort (and eliminated from the pre-COVID cohort) because there were fewer assessments in the COVID cohort. In cases where multiple non-admission assessments were available for an individual, the most recent assessment was kept.

### Quality indicators

The following 16 prevalence-based QIs were used: hospitalizations in the last 90 days of life, emergency department visits in the last 90 days of life, falls, disruptive or intense daily pain, severe or excruciating pain that is inadequately controlled by medication, constipation, shortness of breath at rest, shortness of breath upon exertion, caregiver distress, negative mood, no advance directive, stasis/pressure ulcers, delirium-like syndrome, nausea or vomiting, and sleep problems. The QIs reflect a number of domains of PEoLC including structure and processes, physical, psychological and psychiatric, ethical and legal aspects of care [34]. These were selected based on an extensive literature review including a recent published study of validated QIs using the interRAI PC [33], and the limitations of available interRAI PC data elements of which several are endorsed by Health Quality Ontario as indicators of quality PEoLC [10, 11]. These QIs formed the basis for evaluating the quality of PEoLC for home care clients and met the criteria of being measurable, reflecting broadly the domains of the NCP framework [34], and in use to some extent in quality improvement and research initiatives. The QIs were defined using available interRAI PC data elements (Suppl 1) and based on definitions reported by Guthrie et al. [33].

### Statistical analyses

All statistical analyses were completed on a remote secure server. Statistical analyses included descriptive statistics, where means and standard deviation (SD) values for continuous variables and percentages for categories were reported for covariates. Prior to examining differences in QI rates, propensity score matching was used to create matched cohorts based on demographic variables and other key covariates (Suppl 2) [35]. Following propensity score matching, the QIs were calculated and the differences in QI rates between cohorts were analyzed using a chi-squared test (= z test of 2 proportions) and effect size. Odds ratios (ORs) and effect sizes were generated to help in judging the clinical significance of group differences, as these measures are not driven by sample size [36]. All statistical analyses were completed using R Version 4.1.2. A two-tailed alpha level of 0.05 was used to identify statistically significant differences.

The two cohorts were matched on the following 21 covariates (Suppl 2): sex, age, Changes in Health, End-Stage Disease and Signs and Symptoms (CHESS) score [37], marital status, Local Health Integrated Network (LHIN) identifier, living arrangement, time since last hospital stay, number of days and total minutes in last week of formal care (e.g., home health aides, home nurse), individual instrumental activities of daily living (e.g., meal preparation, ordinary housework, managing medications) and individual activities of daily living (e.g., bathing, personal hygiene, walking, locomotion, transfer toilet, toilet use, and eating). The CHESS detects frailty and health instability, with higher scores indicating greater instability. The LHIN identifier reflects the geographic region where home care is coordinated and funded. These covariates were identified based on current literature and the availability of interRAI data elements.

As part of the statistical analysis, propensity score matching was used to reduce the effect of confounding variables between the two cohorts, thereby creating a more equivalent comparison cohort [35]. Acceptability of the matching procedure was based on falling below the recommended threshold of 0.1 of the standardized mean difference (SMD) for each covariate [38]. Nearest neighbour matching (NNM) was chosen as the primary propensity score method and a sensitivity analysis was used to explore weighted and overlap weighted propensity score methods. NNM is the most common form of matching used in propensity score analysis [39]. It is a matching method based on a distance measure and can employ a caliper. The method involves random selection of a treatment unit (COVID assessment) which is then matched to a control unit (pre-COVID assessment) that falls within the caliper and the process stops when all treatment units are matched. If a treatment unit is not matched, it is dropped.

The distance (caliper) was set to 0.2 for this study, which has been shown in prior research to be suitable for a variety of settings and regarded as optimal, thus it was used for this study [40]. The effect of using a larger caliper, or not using a caliper at all, would be to increase the sample size, but this typically results in poorer covariate balance. Matching without replacement (also called 1:1 matching) was another methodological decision made where each control unit is only matched to one treatment unit [41].

In terms of the additional propensity score methods, weights are employed to reflect the importance assigned to propensity scores and covariate balance during matching. The aim is to use more of the sample using multiple controls per treatment unit and weighted composites of controls, unlike NNM which uses paired-matching. Matching weight and overlapping weight were the two weighted methods used. The matching weight method was proposed by Li and Green [42] and is comparable to one-to-one pair matching without replacing; however, instead of discarding unmatched treatment units, no unit is ever rejected entirely but instead is down-weighted so that multiple controls can be matched with the weight distributed among these units where a fraction of the unit is contributing. As units are weighted so that they contribute less to the sample than with unweighted units, the effective sample size may be lower than with paired matched [43]. The overlapping weight method proposed by Li et al. [44] is another weighting method that matches on propensity score as well as the covariates. The method works similarly to the matching weight method, but the weights are based on both the propensity score and covariates which results in the exact balance on the means of all the covariates included. As a result, units are automatically down weighted with extreme propensity scores. The best practice for selecting propensity score methods is to try multiple methods and explore all those that meet the pre-established criteria for acceptability, as there is no universally superior method [43, 45]. In this study, the pre-established criteria for acceptability were adequate covariate balance. If the methods met these two criteria, they were considered equally valid for use in the analysis [43].

## Results

In creating the pre-COVID and COVID cohorts, 6,346 and 3,231 unique assessments respectively, met the criteria (i.e., not an admission assessment, non-duplicate assessment, and within the pre-established timeframe) to be included in the final cohorts. For the pre-COVID cohort, the breakdown of records were 83.8% (n = 5321) routine reassessments, 7.3% (n = 466) return assessments, and 8.8% (n = 559) significant change in status reassessments. For the COVID cohort, the breakdown of records were 83.8% (n = 2709) routine reassessments, 6.2% (n = 201) return assessments, and 9.9% (n = 321) significant change in status reassessments.

Any records with missing data on the covariates were excluded prior to propensity score analysis which included 214 records from the pre-COVID cohort and 159 records from the COVID cohort. There were no patterns as to the missing data being related to a particular home care jurisdiction or reason for assessment. Multiple imputation was not used as the number of observations fell well below the 5% threshold commonly cited in literature [46].

When calculating the outcomes (QI rates), missing data were only encountered for the pain measure used to calculate prevalence of severe or excruciating daily pain (n = 6) and prevalence of severe or excruciating pain that is not controlled by therapeutic regimen in the pre-COVID cohort (n = 6) after matching the cohorts using NNM. These records were eliminated for the respective QI rate applicable as the data was incomplete. After matching the cohorts using NNM, 3.37% of the pre-COVID and 4.92% of the COVID cohorts could not be matched.

### Propensity score analysis

Propensity score analysis was used to match the cohorts on 21 covariates. *After running the propensity score analysis, all three propensity score analysis methods (NNM, weights and overlapping weights) produced acceptable SMDs (< 0.1) for each of the covariates*. Table 1 provides the results from the statistical analysis comparing the QI rates for the two groups, using the NNM method in the propensity score analysis.

SMDs for the NNM ranged from 0.002 to 0.062 with the cohort size as 2,479 for both pre-COVID and COVID cohorts. The SMDs for the two other propensity score methods were even smaller than with the NNM methods. For the subsequent analysis, it was decided to use the NNM propensity score method as the primary approach for presenting the results as it retained the largest sample size and met the 0.1 SMD threshold, and the other two propensity score methods were explored in a sensitivity analysis.

Several QIs were found to have statistically-significant differences between the pre-COVID and COVID cohorts (prevalence of severe or excruciating daily pain, caregiver distress, negative mood, delirium-like syndrome, and nausea or vomiting), though effect sizes from the statistical analysis were small and thus, the differences were not clinically meaningful. In considering the effects sizes for each QI difference, they were all found to be well below the threshold for clinical significance (0.2) except for prevalence of a delirium-like syndrome which would still be considered a small difference based on Cohen’s guidelines for interpreting effect size (0.2-0.499) [47]. For example, the decrease for depressed mood pre-COVID to during COVID would not be considered a clinically significant finding given the small effect size (effect size= -0.17). When running the sensitivity analysis to examine QI rates and measures of statistical analysis of difference using the two alternative propensity score analysis methods (weights, overlapping weights), these differences in QI rates were found to be no longer statistically significant in the weights method. Importantly, all three propensity score methods showed no clinically meaningful differences between the cohorts (e.g., effect size for prevalence of negative mood: -0.15 and prevalence of nausea or vomiting: -0.10 for the weights method and effect size for prevalence of negative mood: -0.16 for the overlap weights method).

The cohorts were in agreement with the lowest and highest prevalence rates. The lowest prevalence rates included the following QIs: prevalence of a delirium-like syndrome 5.17% (pre-COVID) and 3.32% (COVID), prevalence of severe or excruciating pain that is not controlled by therapeutic regimen 6.75% (pre-COVID) and 5.81% (COVID), prevalence of negative mood 8.43% (pre-COVID) and 6.21% (COVID), and prevalence of ulcers 12.53% (pre- COVID) and 11.74% (COVID). The highest prevalence rates included the following QIs: prevalence of shortness of breath when performing moderate/normal day-to-day activities 49.50% (pre-COVID) and 51.27% (COVID), prevalence of no advance directives 44.90% (pre-COVID) and 46.43% (COVID), prevalence of fatigue 40.74% (pre-COVID) and 41.51% (COVID) and prevalence of sleep problems 35.74% (pre-COVID) and 35.26% (COVID).

**Table 1:** Propensity Score Matching Method 1 (NNM): n = 2479 each (Precovid, Covid)

QI Description	Pre-COVID Cohort (%) n = 2479	COVID Cohort (%) n = 2479	Statistical Analysis of Difference				
			**Χ** ^**2**^ **(z)** ^**a**^	**QI Diff** **(95% CI)** ^**b**^	**Χ** ^**2**^ **(z)** ***p-value*** ^**b**^	**OR** ***(p-value)*** **(95% CI)** ^**c**^	**Effect Size** ^d^
Prevalence of severe or excruciating daily pain	16.70	14.40	4.98 (2.23)	-0.02 (-0.005, -0.08)	**0.03**	0.86 (0.03) (0.76, 0.98)	-0.08
Prevalence of severe or excruciating pain that is not controlled by therapeutic regimen	6.75	5.81	1.87 (1.37)	-0.009 (-0.10, 0.02)	0.17	0.86 (0.17) (0.69, 1.07)	-0.08
Prevalence of emergency department visits	20.40	18.26	3.51 (1.87)	-0.02 (-0.07, 0.002)	0.06	0.90 (0.06) (0.80, 1.01)	-0.06
Prevalence of hospital admissions	24.89	22.71	3.23 (1.80)	-0.02 (-0.06, 0.003)	0.07	0.91 (0.07) (0.83, 1.01)	-0.05
Prevalence of falls	24.06	23.00	0.73 (0.85)	-0.01 (-0.05, 0.02)	0.39	0.96 (0.39) (0.86, 1.06)	-0.02
Prevalence of constipation	18.15	16.42	2.61 (1.62)	-0.02 (-0.07, 0.007)	0.11	0.90 (0.11) (0.80, 1.02)	-0.06
Prevalence of shortness of breath at rest	15.21	15.89	0.44 (0.66)	0.007 (-0.03, 0.05)	0.51	1.05 (0.51) (0.92, 1.19)	0.03
Prevalence of shortness of breath when performing moderate/normal day-to-day activities	49.50	51.27	1.56 (1.25)	0.02 (-0.01, 0.05)	0.21	1.04 (0.21) (0.98, 1.09)	0.02
Prevalence of caregiver distress	24.10	21.36	5.26 (2.29)	-0.03 (-0.005, -0.07)	**0.02**	0.89 (0.02) (0.80, 0.98)	-0.06
Prevalence of negative mood	8.43	6.21	8.99 (3.00)	-0.02 (-0.03, -0.14)	**0.003**	0.74 (0.003) (0.60, 0.90)	-0.17
Prevalence of no advance directives	44.90	46.43	1.17 (1.08)	0.02 (-0.01, 0.04)	0.28	1.03 (0.28) (0.97, 1.10)	0.02
Prevalence of ulcers	12.53	11.74	0.71 (0.84)	-0.008 (-0.06, 0.03)	0.40	0.94 (0.40) (0.81, 1.09)	-0.03
Prevalence of a delirium-like syndrome	5.17	3.32	10.43 (3.23)	-0.02 (-0.04, -0.18)	**0.001**	0.64 (0.001) (0.49, 0.84)	-0.25
Prevalence of nausea or vomiting	19.65	17.43	4.04 (2.01)	-0.02 (-0.0003, -0.07)	**0.04**	0.89 (0.04) (0.79, 1.00)	-0.06
Prevalence of fatigue	40.74	41.51	0.30 (0.55)	0.008 (-0.02, 0.04)	0.58	1.02 (0.58) (0.95, 1.09)	0.01
Prevalence of sleep problems	35.74	35.26	0.13 (0.36)	-0.005 (-0.03, 0.02)	0.72	0.99 (0.72) (0.92, 1.06)	-0.01

^a^ z2 = χ2, used frequencies from 2 × 2 table obtained using proportions, χ2 obtained from R (see note b):

^b^ QI Diff = COVID-Pre-COVID, 95% obtained from R [res<-prop.test(x = c(407, 2072), n = c(857, 4101)) c OR stats from online calc: https://www.medcalc.org/calc/relative_risk.php

^d^*d* = LogOddsRatio x (√3/π) = Ln(OR) x 0.551328

## Discussion

This study is the first study to examine differences in QI rates for home care clients receiving PEoLC before and during COVID in Ontario. It appeared that QI rates did not change over the course of the pandemic in this population. Several QIs were found to have statistically significant differences between the pre-COVID and COVID cohorts, though effect sizes from the statistical analysis were small and thus, the differences were not clinically meaningful. There are a number of other considerations (e.g., temporal variation, interpreting single QI rates and methodological choices) that warrant exploration in order to interpret these findings.

These QIs and the comparison of them across the cohorts should be interpreted with caution for several reasons. Firstly, any single number or QI rate cannot directly indicate that poor quality of care was delivered [48, 49]. Rather, QIs serve as a proxy to the quality of care delivered and can provide valuable information on the state of PEoLC [50, 51]. Additionally, a single QI rate captures the effect of a range of service/quality-related changes, and those changes cannot be understood from the analysis of a single number. Additionally, the temporal variation in rates is unknown and needs to be explored as there may be statistically significant differences in the rates over time within each cohort. Though the pre-COVID cohort provided a suitable comparator cohort, variation of these rates over time is largely unknown and statistical differences between the cohorts may reflect natural variation in QI rates over time (not cohort differences).

There were statistically significant differences in a few QIs from the first propensity score analysis method, NNM. However, these few statistically significant differences were inconsistent across the three propensity score methods (all of which met acceptability criteria). Moreover, there were no clinically meaningful differences between the cohorts when examining the effect sizes. These discrepancies can be explained by methodological issues such as different sample sizes and the resulting differences in the assessments kept when matching on covariates. NNM was thus used for the primary analysis, and the weighted methods were explored as part of a sensitivity analysis. While there are advocates for different propensity score methods, the empirical evidence for any singular method is varied and does not often achieve better performance despite the theoretical or conceptual defence [43].

In interpreting the results of this study, a *p*-value is not sufficient alone to interpret the differences between these QI rates as it does not account for sample size [52]. Subsequently, effect sizes and odds ratios were explored and aided in interpreting the results of this study. Additionally, *p*-values were not adjusted for because there were no consistent statistically significant and clinically meaningful results, thus the question of whether significant results were due to high error rates arising from multiple comparisons did not arise.

The severity of outcome can also be a helpful descriptor when interpreting the magnitude of effect size. Caution is recommended in using a generic interpretation guidelines when considering the effect sizes of different outcomes [53]. For example, some QIs measure more severe outcomes, such as severe pain uncontrolled by medications or caregiver distress and clinically speaking, may be more meaningful or amenable to change. Therefore, each of the QIs should not be considered of equal weight in comparing them among fellow QIs.

While there may not have been clinically meaningful differences in any QI rates, it is also important to note that there were some concerning rates regardless of time period for the prevalence of shortness of breath when performing moderate/normal day-to-day activities, prevalence of no advance directives and prevalence of sleep problems QIs. While the QIs cannot definitely determine whether poor quality of care was delivered, they can provide an indication of areas where further investigation into care quality/practices is warranted concern [49]. As previously mentioned, the home care sector has been recognized as not optimally organized and funded to support quality PEoLC long before the COVID-19 pandemic and high QI rates may reflect this long-standing problem [10, 11, 24]. The fact that several of these QIs were experienced by almost half of the population should warrant further attention as these QIs reflect outcomes of care that may be amendable to change.

### Strengths and limitations

There are several strengths in relation to this study. As to the author’s knowledge, this study is of the first to examine the use of QIs for home-based PEoLC using interRAI data during the COVID-19 pandemic and to compare the QI rates between time periods. These QIs are based on the interRAI PC, a standardized clinical assessment instrument used in Ontario with established reliability and validity [30]. The strength of many of the QIs used in this study is that they focus on symptoms and patient-reported outcomes which are less frequently reported in the literature [49, 54, 55]. Finally, a rigorous cohort definition and statistical approach were adopted, where propensity score matching was used to reduce the influence of numerous covariates on the QI rates, multiple matching methods (e.g., NNM, weights, overlap weights) were explored to look at the changes in QI rates over time and multiple statistical measures were compared (e.g., chi-square test, odds ratio, effect size).

The study has several limitations, particularly by means of timeline, sample size sufficiency, ascertainment bias (e.g., only those with a follow-up assessment were included, and during COVID fewer patients may have received a PC assessment or follow-up assessment), power and the types of QIs able to be examined. One major limitation is in comparing QI rates at different timepoints without any sense of temporal variation in the measure. Given the uncertainties around how to define QIs, how they vary over time, and how or if they respond to practice changes, a sample size was not hypothesized in relation to an expected change in effect size. For QIs such as emergency department visits or hospitalizations, there may be limitations as they do not account for personal preferences and the ability to examine planned or unplanned service use that could be deemed appropriate. In terms of methodology, while state-of-the art techniques were used to balance the cohorts (PSM), this technique could only capture covariates available in the interRAI PC. Therefore, other important covariates such as month (e.g., seasonality of flu), income or ethnicity may be missing [6]. Additionally, this study only examined home care clients who received PEoLC and does not account for those who were unable to receive care. A further limitation may exist in relation to power; detecting a difference in proportions requires a large sample size. If the largest QI difference we observed in this study (3%) is regarded as clinically-meaningful, to detect this with 80% power and 5% alpha requires a sample size of approximately 3,000 per cohort [56], which exceeds the sample size we had using the NNM approach. Finally, there are no agreed-upon QIs for palliative and end-of-life home care in Canada. There is work underway [33, 51]; however, not definitive yet and thus the QIs used in this study may not be comprehensive in what are adopted for quality in the future.

## Conclusion

This retrospective population-based cohort study compared QI rates for home care clients designated to receiving PEoLC prior to and after the onset of the COVID-19 pandemic. The sixteen QIs examined in this study did not change over the course of the pandemic in this population. While several QIs were found to have statistically significant differences between the pre-COVID and COVID cohorts, the differences were not clinically meaningful. Future work should be directed to examining variation in these QI rates and their expected stability over similar time increments, risk-adjusting the QI rates for further comparison among jurisdictions, provinces and countries and establishing benchmarks or thresholds to determine acceptable rates of different QIs.

### Electronic supplementary material

Below is the link to the electronic supplementary material.

Supplementary Material 1

Supplementary Material 2

## Data Availability

The data that support the findings of this study may be available from interRAI, but restrictions apply to the availability of these data, which were used under license for the current study, and are not publicly available.
