# Supplementary material for: Comparing quality indicator rates for home care clients receiving palliative and end-of-life care before and during the Covid-19 pandemic
Source: BMC Palliat Care. 2024 Jan 5;23:11. doi: 10.1186/s12904-023-01336-9 (PMC10768311; doi:10.1186/s12904-023-01336-9)
Supplement: Supplementary file 1 — Supplementary Material 1 [file 12904_2023_1336_MOESM1_ESM.docx]

Quality Indicator Definitions and Calculations

| **Title** | **Numerator and**  **Denominator Definitions** |
| --- | --- |
| Prevalence of severe or excruciating daily pain | **N:** Client has daily pain that is severe or excruciating  **D:** All clients who have had a  re-assessment |
| Prevalence of severe or excruciating pain that is not controlled by therapeutic regimen | **N:** Client has daily pain that is severe or excruciating  and pain is not controlled by current therapeutic regimen  **D:** All clients who have had a  re-assessment |
| Prevalence of emergency department visits | **N:** Client experiences an emergency department visit without an overnight stay  **D:** All clients who have a  re-assessment and who are not imminently dying |
| Prevalence of hospital admissions | **N:** Client experiences at least one hospital admission with an overnight stay  **D:** All clients who have a  re-assessment and who are not imminently dying |
| Prevalence of falls | **N:** Client experienced one or more falls within the last 90 days  **D:** All clients who have a  re-assessment and are not completely dependent in bed mobility |
| Prevalence of constipation | **N:** Client experiences constipation, no bowel movement in three days or difficult passage of hard stool  **D:** All clients who have had a  re-assessment |
| Prevalence of shortness of breath at rest | **N:** Client experiences shortness of breath at rest  **D:** All clients who have had a  re-assessment |
| Prevalence of shortness of breath when performing moderate/normal day-to-day activities | **N:** Client experiences shortness of breath when performing moderate or normal day-to-day activities  **D:** All clients who have had a  re-assessment |
| Prevalence of caregiver distress | **N:** Client’s primary caregiver expresses feelings of distress, anger, or depression  **D:** All clients on re-assessment with a primary caregiver |
| Prevalence of negative mood | **N:** Client has a DRS score of ≥4  **D:** All clients who have had a  re-assessment |
| Prevalence of no advance directives | **N:** Client does not have an advance directive in place for any of the following: not resuscitating, not intubating, not hospitalizing, sending to emergency department, not tube feedback, and medication restriction  **D:** All clients who have had a  re-assessment |
| Prevalence of ulcers | **N:** Client has a pressure ulcer or another type of ulcer  **D:** All clients who have a  re-assessment who are not imminently dying |
| Prevalence of a delirium-like syndrome | **N:** Client experiences an acute change in mental status and also experiences at least one of the following: Fluctuating state of consciousness, Mental functioning varies over the course of the day or Hallucinations  **D:** All clients who have a re-assessment who are not imminently dying |
| Prevalence of nausea or vomiting | **N:** Client experiences vomiting or nausea  **D:** All clients who have had a  re-assessment |
| Prevalence of fatigue | **N:** Client experiences an inability to complete normal day-to-day activities due to fatigue  **D:** All clients who have had a  re-assessment |
| Prevalence of sleep problems | **N:** Client has difficulty falling asleep, staying asleep, waking up too early, experiences restlessness or experiences non-restful sleep  **D:** All clients who have had a  re-assessment |
