# Supplementary material for: Comparing quality indicator rates for home care clients receiving palliative and end-of-life care before and during the Covid-19 pandemic
Source: BMC Palliat Care. 2024 Jan 5;23:11. doi: 10.1186/s12904-023-01336-9 (PMC10768311; doi:10.1186/s12904-023-01336-9)
Supplement: Supplementary file 2 — Supplementary Material 2 [file 12904_2023_1336_MOESM2_ESM.docx]

Covariates for Propensity Score Analysis and Definitions

| **Item from the interRAI PC*** | **Definition/description** | **Response options** |
| --- | --- | --- |
| Sex |  | 1=Male  2=Female |
| Age in years |  |  |
| Marital status |  | 1=Never married  2=Married  3=Partner/significant other  4=Widowed  5=Separated  6=Divorced |
| Home Care Jurisdiction Identifier | Geographic region where home care is  coordinated and funded | Coded as an acronym (e.g.,  CHA – Champlain, NW – North  West) |
| Living arrangement | Who did the person live with at the time  of the assessment | 1=Alone  2=With spouse/partner only  3=With spouse/partner and  other(s)  4=With child (not  spouse/partner)  5=With parent(s) or guardian(s)  6=With sibling(s)  7=With other relative(s)  8=With nonrelative(s) |
| Time since last hospital stay | Number of days since the last hospital  admission | 0=None within 90 days  1=31 to 90 days ago  2=15 to 30 days ago  3=8 to 14 days ago  4=In the last 7 days  5=Now in hospital |
| Instrumental Activities of Daily Living Self-Performance (e.g., meal preparation, ordinary housework, managing medications) | Ability to perform IADLs independently | 0=Independent  1=Set-up help only  2=Supervision  3=Limited assistance  4=Extensive assistance  5=Maximal assistance  6=Total dependence  8=Activity did not occur |
| Activities of Daily Living Self-Performance: bathing, personal hygiene, walking, locomotion, transfer toilet, toilet use, eating |  | 0=Independent  1=Set-up help only  2=Supervision  3=Limited assistance  4=Extensive assistance  5=Maximal assistance  6=Total dependence  8=Activity did not occur |
| Receipt of formal care from a home health aide |  | Number of days that care was  received in the last 7 days |
| Formal care: Home health aides: total minutes in last week | Continuous variable (total minutes in last  week) |  |
| Formal care: Home nurse: number of days | Continuous variable (number of days) |  |
| Formal care: Home nurse: total minutes in last week | Continuous variable (total minutes in last  week) |  |
| Changes in Health, End-Stage Disease and Signs and Symptoms (CHESS) score | Health index scale that detects frailty and  health instability | 0=No symptoms  1=Minimal health instability  2=Low health instability  3=Moderate health instability  4=High health instability |

*The variables included here are part of the interRAI PC instrument. The instrument is copyrighted by interRAI and as such, only variables used are shared. For more information about the tool, visit: https://catalog.interrai.org/content/interrai-palliative-care-pc-assessment-form-and-user%E2%80%99s-manual-standard-english-edition-912
